# Emergency Standing Laparoscopic Treatment of Uncontrolled Post-Castration Hemorrhage in Two Geldings

**DOI:** 10.3390/ani14152252

**Published:** 2024-08-02

**Authors:** Barbara Delvescovo, Rebecca McOnie, Garett Pearson, Brenna Pugliese, Eileen S. Hackett

**Affiliations:** Department of Clinical Sciences, Cornell University College of Veterinary Medicine, Ithaca, NY 14853, USA; bd382@cornell.edu (B.D.); gbp34@cornell.edu (G.P.); brpuglie@ncsu.edu (B.P.); esh82@cornell.edu (E.S.H.)

**Keywords:** castration, horse, hemorrhage, laparoscopy, testicular artery, standing

## Abstract

**Simple Summary:**

Equine castration is a commonly performed veterinary surgical procedure in young colts. The procedure involves surgical removal of both testicles by transection of the spermatic cord. One complication of the procedure includes persistent hemorrhage from blood vessels within the spermatic cord and surrounding tissues. In severe cases, the hemorrhage can be life-threatening. Common treatments include packing gauze within the scrotum or attempted direct ligation of the blood vessels. Retrieval of a retracted spermatic cord is very difficult in the standing horse, and general anesthesia may be required. The risks of general anesthesia, including anesthetic recovery, could be increased in cases of hemorrhagic hypovolemia. The extensive tissue manipulation necessary for spermatic cord retrieval within the open castration site can result in further contamination. Minimally invasive techniques from abdominal approaches have been described to reduce these risks. Here, we describe a standing laparoscopic approach to cord ligation in two horses as an emergency procedure for marked hemorrhage post-castration. The feasibility and efficiency of this approach are demonstrated.

**Abstract:**

Background: Persistent hemorrhage of testicular vessels is a potentially life-threatening complication of equine castration. Frequently, general anesthesia is required to retrieve and ligate the bleeding vasculature when standing wound packing and retrieval of the spermatic cord are unsuccessful. We propose standing laparoscopic ligation of the testicular arteries via the paralumbar fossa as a rapid, effective means of halting hemorrhage while avoiding castration site trauma as well as the cardiovascular and recovery risks of general anesthesia. Methods: Two geldings, 6 and 9 months old, presented for emergency treatment of severe post-castration hemorrhage of 10 and 24 h durations, respectively. Both geldings underwent standing laparoscopy under light sedation and the testicular vessels were ligated using a bipolar vessel-sealing device. Results: Testicular vessel sealing was successfully performed in both geldings by standing laparoscopy and resulted in immediate cessation of hemorrhage. In one case, a left paralumbar fossa approach allowed coagulation of both the left and right spermatic vessels. The procedure time was 25 and 35 min. No complications occurred, and both geldings recovered uneventfully. Conclusions: Standing, laparoscopic ligation of the testicular arteries is a feasible emergency treatment in young geldings and can be applied in cases of uncontrolled post-castration hemorrhage.

## 1. Introduction

Equine castration is a commonly performed field surgery that leaves a scrotal wound to heal by second intention. Persistent hemorrhage post-castration is an uncommon complication with a recently reported prevalence of 1.8–2.4% [1,2,3,4]. Transient, mild bleeding during surgical incision of the skin and subcutaneous tissues as well as immediately after transection of the spermatic cord or removal of the emasculator is normal. However, fast drips (>1 drop/second) or a stream of blood for more than 15–30 min is an emergency that requires prompt intervention [2,5,6]. 

Various factors can contribute to inadequate hemostasis during the procedure: inappropriate technique during ligature application, tearing of the cremaster muscle, faulty emasculator application, equipment malfunction and, less commonly, individual pathologies affecting the ability of blood to coagulate. In young horses, the tendency towards variable positioning of the testicle between the external inguinal ring and scrotum might increase the risk of some complications due to impaired surgical access to the spermatic cord. The most common source of significant persistent postoperative hemorrhage is the testicular artery, but bleeding can also occur from the testicular vein, cremaster or dartos muscles, as well as superficial skin and subcutaneous vessels [7]. Hemorrhage from the testicular artery can be fatal if persistent and ineffectively addressed. Common interventions for hemostasis include scrotal wound packing it with sponges to compress the bleeding vessel and promote clot formation, and ligation of the cord when it can be retrieved on deep incisional exploration [5]. In many cases, general anesthesia (GA) and dorsal recumbency are required to successfully ligate the spermatic cord or adequately pack the wound to stem hemorrhage [5]. 

Laparoscopic approaches offer the advantage of minimizing castration site trauma and improving cord component access to guide ligation [5,8,9]. Standing laparoscopy offers the additional advantage of avoiding general anesthesia and its associated risks [10,11]. Earlier reports of horses treated with standing, laparoscopic testicular artery ligation reveal its useful application in a case of chronic hemoabdomen and acute post-castration hemorrhage [8,9,12,13]. The objective of this report is to describe the application and outcomes of a standing, sedated laparoscopic approach for emergency treatment of persistent and life-threatening hemorrhage in two colts following routine field castration. Additionally, we describe a unilateral, paralumbar fossa approach for ligation of both testicular arteries. 

## 2. Materials and Methods: Case Details

Case information: Case 1, a six-month-old appaloosa gelding (171 kg), and case 2, a 9-month-old mixed breed gelding (300 kg), were presented on referral for treatment of persistent, moderate to severe post-castration hemorrhage, of 10 and 24 h durations, respectively. Both geldings underwent routine castration in the field. Management of the persistent bleeding prior to referral included attempted cord ligation and scrotal wound packing with sponges. In both cases, these attempts were unsuccessful at stopping or significantly reducing the hemorrhage. 

Clinical findings: On presentation, both geldings had evidence of significant blood loss and had active, moderate unilateral hemorrhage from their scrotal wounds. In both cases, the hemorrhage was presumed to be left-sided. Clinical signs of hemorrhagic shock characterized by tachycardia (68 bpm and 116 bpm), pale mucous membranes and delayed jugular refill time were observed in both cases. Additionally, point-of-care bloodwork revealed moderate hyperlactatemia (3.37 mmol/L and 3.4 mmol/L) (reference range < 2 mmol/L), anemia (PCV 26% and 25%) (reference range 28–53%) and hypoproteinemia (4.6 g/dL and 3.8 g/dL) (reference range 6.0–8.1 g/dL). The above values in parentheses refer to case 1 and case 2, respectively.

Therapeutic intervention: Medical stabilization was immediately initiated and included placement of an intravenous jugular catheter, administration of balanced crystalloid fluids (plasmalyte 20 mL/kg) and broad-spectrum injectable antimicrobials (Potassium Penicillin 22,000 IU/kg IV (Athenex Pharmaceuticals, Schaumburg, IL, USA) or Procaine Penicillin IM (Norocillin^®^, Norbrook Laboratories, Norbrook Laboratories Limited Newry, Co., Down, Northern Ireland) and Gentamicin Sulfate 6.6 mg/kg IV (Covertus, Portland, ME, USA)). Analgesia was provided with flunixin meglumine (1.1 mg/kg IV; Banamine^®^ Merck & Co., Inc., Rahway, NJ, USA) and morphine bolus (0.1 mg/kg slow IV; Morphine Sulfate injection, Hikma Pharmaceuticals USA Inc., Cherry Hill, NJ, USA) in case 2. Simultaneously, the geldings were readied for surgical intervention by clipping and aseptic preparation of the paralumbar fossae. The horses were restrained in stocks and underwent standing, sedated left-flank laparoscopy with similar approaches. Detomidine hydrochloride (0.006 mg/kg IV; Dormosedan^®^ Zoetis Inc., Kalamazoo, MI, USA) was administered; this dose was sufficient to maintain an appropriate plane of anesthesia for the brief procedure in case 1. Following the initial detomidine bolus, an infusion of detomidine (0.1–0.6 mcg/kg/min IV) was administered to effect in case 2. After clipping, aseptic preparation and draping, 5 mL of 2% mepivacaine hydrochloride (Carbocaine^®^ Zoetis Inc., Kalamazoo, MI, USA) was administered subcutaneously and intramuscularly at the sites of intended left paralumbar fossa incisions. 

**Figure 1:**
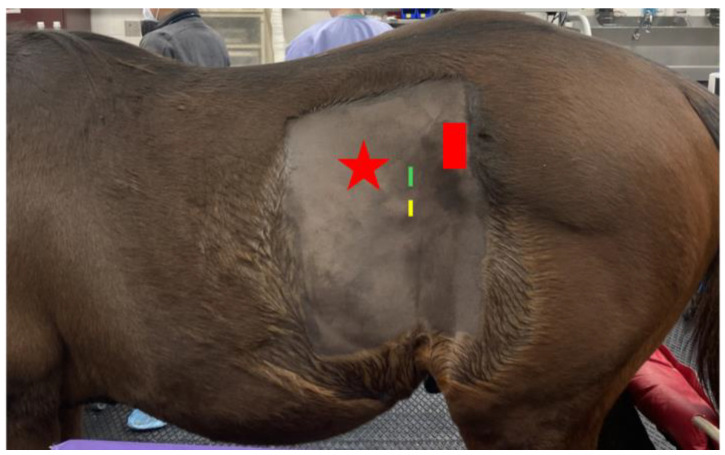
A standing, sedated horse restrained in stocks with a clipped left flank. The red star represents the location of the last rib, and the red rectangle indicates the tuber coxa. Laparoscopic portal locations are depicted by the green and yellow lines. The laparoscope was inserted into the portal identified by the green line, and the bipolar vessel-sealing device was inserted through the portal identified by the yellow line.

**Figure 2:**
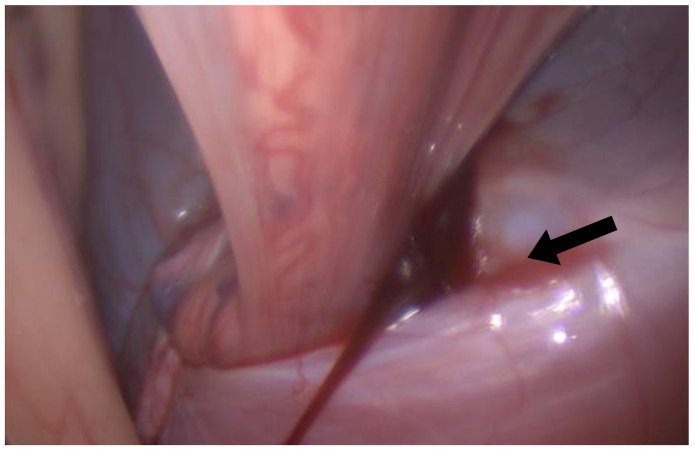
Laparoscopic image of the left internal inguinal ring with a visible clot (black arrow).

**Figure 3:**
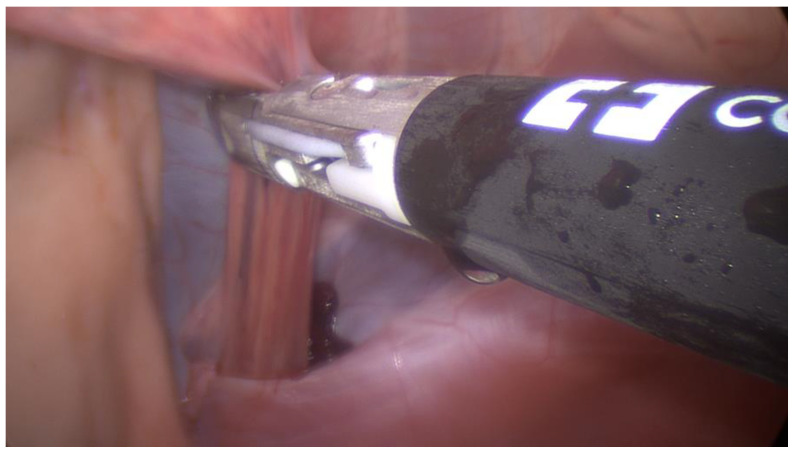
Laparoscopic image of the left internal inguinal ring during application of the bipolar vessel-sealing device.

The first portal was positioned standardly in the left paralumbar fossa, dorsal to the internal abdominal oblique crus and halfway between the last rib and tuber coxa (Figure 1). A rigid, 30-degree endoscope was used to confirm cannula placement in the abdomen, and peritoneal insufflation with carbon dioxide to 12 mmHg was achieved (Karl Storz Veterinary Endoscopy, Tuttlingen, Germany). A second port was made about 5 cm ventral to the first for introduction of the bipolar vessel-sealing device (5 mm, 37 cm Covidien Ligasure; Figure 1). The left internal inguinal ring was identified. In both cases, the spermatic cords were visible within the inguinal canal and not retracted into the abdomen; a large clot was visible near the left internal inguinal ring surrounding the spermatic vasculature (Figure 2). In case 1, mild intra-abdominal hemorrhage was also noted. Local anesthetic (5 mL of 2% lidocaine injectable solution, Covetrus, Portland, ME, USA) was administered as a splash block over all spermatic cord components. The testicular artery and other cord components were cauterized using the Ligasure (Figure 3) with the affected sides (left) being double-ligated (Video S1) and having no tissue transection performed. External scrotal hemorrhage stopped immediately after ligation of the affected testicular artery in both horses.

A blunt probe (Palpation probe, Karl Storz Veterinary Endoscopy, Tuttlingen, Germany) was then used to elevate the descending colon and view the contralateral (right) inguinal ring and cord components. Both horses also had small hematomas in the right inguinal canal, protruding slightly through the internal inguinal rings; therefore, the Ligasure was deployed across the right cord components as a precaution (Video S1). In the smaller horse (case 1, 171 kg), hemostasis of the right-sided cord components was achieved with the laparoscope and Ligasure extending across the abdomen from the left portals. However, in the larger horse (case 2, 300 kg), this was not possible due to the Ligasure length, and portals were similarly created on the right for ipsilateral ligation of the testicular vessels using the Ligasure.

The total surgical time for case 1 was 25 min and 35 min for case 2. The geldings remained comfortable and tolerant during the procedure. Both horses recovered well from the procedure. They were each administered intravenous crystalloid fluids until their lactate normalized (at about 6–12 h post-admission). Case 2 was additionally treated during the procedure with a 3 L whole-blood transfusion and a single dose of aminocaproic acid (40 mg/kg IV; American Regent, Shirley, NY, USA). The blood transfusion was administered due to clinical concerns, including marked tachycardia, for diminished oxygen carrying capacity, whereas the aminocaproic acid was administered to decrease the risk of further blood loss as preparations for the procedure were performed. Postoperative injectable flunixin meglumine and antimicrobials were continued for 3 and 4 days of hospitalization, for cases 1 and 2, respectively. Then, both geldings were transitioned to trimethoprim-sulfadiazine at 30 mg/kg PO BID (Amneal Pharmaceutical, Bridgewater, NJ, USA) and oral flunixin meglumine at 1.1 mg/kg SID (Banamine^®^ paste, Merck & Co., Inc., Rahway, NJ, USA) for continued treatment at home. Neither of the horses encountered complications during hospitalization or at home. Both patients had a good long-term outcome. 

## 3. Discussion

We report a standing laparoscopic approach that allowed immediate and uncomplicated ligation of the bleeding cord vessels in two cases of acute, life-threatening post-castration hemorrhage. The laparoscopic procedure was successfully and rapidly performed without intra- or postoperative complications. 

When post-castration hemorrhage occurs, attempts to identify and ligate the source of bleeding, either standing or under general anesthesia, are challenging due to tissue swelling and pooling blood. Additionally, there is a significant risk of contaminating inflamed tissues associated with the surgical site, including the subcutaneous tissue, spermatic cord and abdomen. The challenge and potential for an adverse outcome might be elevated when retraction of the spermatic cord is dramatic, making identification and retrieval of a bleeding vessel through a scrotal surgical incision difficult or impossible [14]. In contrast, surgical intervention by an abdominal approach bestows the advantages of enhanced access to a retracted spermatic cord and an aseptic surgical site distant to the scrotum that could limit contamination capable of causing spermatic cord infection or septic peritonitis [14].

Laparoscopic approaches to spermatic cord ligation in cases of post-castration hemorrhage have been reported in dorsally recumbent and in standing sedated horses [8,9,12,13]. Standing laparoscopy has advantages over laparoscopy under GA or an open abdominal approach. The first is in avoiding general anesthesia and the associated depressant cardiovascular effects of recovery risks [10,11]. The risks associated with GA increase in patients that are actively hemorrhaging and cardiovascularly unstable like the cases in the present report. Trendelenburg positioning, needed to effectively see the inguinal rings and spermatic cord components with the horse in dorsal recumbency, increases the patient’s cardiovascular and pulmonary impairment [15]. Excellent observation of and access to the spermatic cord components is facilitated with standing laparoscopy due to maintenance of the normal abdominal viscera topography and the tendency of blood to accumulate ventrally in cases of hemoabdomen.

To the authors’ knowledge, this is the first time that a standing unilateral paralumbar fossa approach is reported for the ligation of bilateral testicular vessels in horses with post-castration hemorrhage. Although the left-sided testicular artery was implicated in active hemorrhage, the contralateral testicular artery was ligated due to the presence of an inguinal canal hematoma and to ensure adequate hemostasis. The narrow abdominal width of the smaller patient (case 1) allowed the advancement of the Ligasure across the abdomen to provide contralateral vessel cauterization, and this technique could be applied in young colts with castration complications. Although unilateral portals for bilateral ovariectomy have been described, the tissue and its attachments for surgical transection in these cases is more mobile and readily accessed based on its dorsal, axial location compared to the spermatic cord components associated with a descended testicle [16]. Since bilateral flank access to spermatic cord ligation with the Ligasure has been reported in horses up to five years of age for castration [17], we anticipate this technique can be reliably used for halting testicular artery hemorrhage in larger horses when portals are made bilaterally. Alternatively, long-handled instrumentation could overcome the length limitation of the Ligasure vessel-sealing device for successful unilateral flank access in a mature horse.

The patient preparation time and procedure duration were short enough that both geldings underwent the procedure while being administered initial fluid resuscitation boluses. This timeframe represents an advantage for promptly treating cases of uncontrolled hemorrhage. The procedure can be carried out with sedation and local anesthetic concurrent with fluid resuscitation and other important therapies, such as blood product transfusions. Although similar standing laparoscopic approaches have been reported, the number of published cases with described outcomes are limited [9,12,13]. The cases described here reinforce the technique’s feasibility with rapid and successful deployment to control post-castration hemorrhage on an emergency basis. The authors recommend standing laparoscopic ligation of spermatic cord components as a suitable treatment for uncontrolled post-castration hemorrhage in horses when the facility, equipment and training allow for it.

## 4. Conclusions

Standing laparoscopic ligation of bleeding testicular vessels is feasible, safe and time-effective in geldings with uncontrolled post-castration hemorrhage. Its application should be strongly considered as emergency treatment in similar cases. Bilateral ligation of testicular vessels from unilateral laparoscopic portals should be attempted in young and small to medium size adult horses.

## Data Availability

The original contributions presented in the study are included in the article/Supplementary Material, further inquiries can be directed to the corresponding author/s.

## Supplementary Materials

The following supporting information can be downloaded at https://www.mdpi.com/article/10.3390/ani14152252/s1, Video S1: Video showing Ligasure device deployed across the right spermatic cord after the descending colon was elevated using a blunt probe to create a path for the laparoscope to see the right inguinal ring and cord components from the left side instrument placement.
